# A spotlight on the surfacing of self-management of employees with diabetes seen by professional nurses in selected occupational health clinics in Cape Town

**DOI:** 10.4102/hsag.v25i0.1430

**Published:** 2020-12-01

**Authors:** Natalie Copeling, Karien Jooste

**Affiliations:** 1 Department of Nursing Science, Faculty of Health and Wellness Science, Cape Peninsula University of Technology, Cape Town, South Africa

**Keywords:** Diabetes mellitus, Employee, Self-management, Occupational healthcare institution, Knowledge

## Abstract

**Background:**

Diabetes is considered one of the largest global health challenges of this century and one of the top 10 causes of death across the world. Studies indicate an increased economic burden in relation to diabetes, not only on government revenue but also within private industries. Exploring the perceptions of employees with diabetes mellitus as related to their self-management practices could assist in encouraging behaviours that are associated with positive management outcomes.

**Aim:**

The purpose of this study was to explore and describe the perceptions of employees with diabetes mellitus, seen by professional nurses in selected occupational health clinics, about the self-management of their disease.

**Methods:**

A qualitative, exploratory, descriptive contextual design was followed through 17 individual interviews with 17 employees working in various industries in Cape Town, using a semi-structured interview schedule. Open coding of the data followed, and four themes emerged. Measures to ensure trustworthiness were also adhered to in the study, and approval for the study was granted.

**Results:**

The study findings gave insight into the manner in which employees perceived their behaviour changes in terms of their self-management practices. Various emotions were expressed by employees relating to the disease as well as the lifestyle adaptations required for self-management thereof. Employees noted that knowledge acquisition and greater understanding of the motivators for lifestyle changes could improve aspects of their day-to-day living as well as the disease outcomes.

**Conclusion:**

Employees expressed an awareness of the changes and lifestyle adaptations needed but found aspects thereof to be an ongoing challenge. The areas of challenge varied amongst employees. A consciousness of the possible consequences of poor self-management practices and self-modification behavior to address these was observed.

## Introduction

The World Health Organisation (WHO) estimates that approximately 422 million adults were living with diabetes mellitus (DM) in 2014 compared to the number in 1980 of 108 million (WHO 2016:6). This illustrated an increase of 314 million adults in 34 years. The WHO defines DM as being a ‘serious threat to population health’. Diabetes is a lifelong chronic disease; however, people with DM can live long and healthy lives. A focus on universal health coverage is essential to ensure that all individuals obtain the health services they need, when and where they need them, without facing financial hardship (WHO 2019). Measures that could support or improve the outcomes of the disease should be explored, including those that can be addressed within an occupational health environment.

A dedicated commitment to achieve universal health coverage is fundamental for those with Type 1 DM, who require daily access to both insulin and blood glucose test strips to survive. This group risks being left behind by global initiatives that fail to consider these particular needs (Klatman, McKee & Ogle 2019). The study by Klatman et al. (2019) indicates that no less-resourced country had even near-complete coverage for insulin, and coverage was worse for test strips.

Nationally, DM accounted for 5.5 million deaths in 2016 in South Africa. Statistics South Africa ranked diabetes as the second most common cause of death in the country in statistics reported in 2016 (Umraw 2018:n.p.). The Society for Endocrinology, Metabolism and Diabetes South Africa (SEMDSA) suggests that this is underestimating the burden of disease because South Africa does not currently have a national diabetes registry (SEMDSA Type 2 Diabetes Guidelines Expert Committee 2017:10). The International Diabetes Federation (IDF) postulates that the cost of the disease globally is one that is affecting governments adversely, especially in low- to middle-income countries, and in order to address it, affordable and cost-appropriate interventions as per best practice principles must be adopted (IDF 2017:7). Studies indicate an increased economic burden in relation to diabetes, not only on the government revenue but also within private industries (American Diabetes Association [ADA] 2017:928; Thiyagarajan & John 2017:45).

The reasons for the ever-increasing prevalence of DM are attributed to an increasing incidence of obesity, the general population growth, ageing populations and urbanisation. A lack of physical activity and a more sedentary lifestyle are cited as causing an increase in the prevalence of Type 2 diabetes (Mohebi et al. 2018:2).

Lifestyle changes may be the most difficult part of a treatment regime for DM. A high percentage of employees with DM may exhibit selective adaptation to medical and dietary lifestyle changes suggested. Self-management of the employee’s diabetes is essential, and it is therefore recommended that dietary education includes promoting a positive attitude towards the disease (Jaworski et al. 2018:163).

The promotion of an employee’s understanding of the disease and the necessity for self-management are imperative in empowering individuals diagnosed with DM. This, in turn, could allow them to make informed decisions about their self-care health practices (Jaworski et al. 2018; Quinn et al. 2011:729).

Much information is available on the proposed management of the disease and the advocated lifestyle changes, but fewer resources are to be found relating to the perceptions of the individuals having to make the changes themselves and the support that employees with DM receive in an occupational health setting (Ahlin & Billhult 2012:42; Mohebi et al. 2018:4).

## Problem statement

The individual’s response to information provided regarding treatment and self-management of the disease, DM, is influenced by the individual’s perceptions relating to the chronic illness. Negative perceptions about the disease could adversely affect the employee’s self-management practices relating to diet, self-care and medication (Ardena et al. 2010:161; Jarab et al. 2018:304).

Occupational health nurses who are members of the South African Society of Occupational Health Nursing Practitioners (SASOHN) meet regularly, and one of the topics of discussion focuses on the need to give attention to healthcare delivery within industries on the management of chronic diseases, including diabetes.

Greater understanding of how DM self-management is perceived by the employee should guide the Occupational Health Nurse Practitioner (OHNP) in the delivery of a more effective and all-encompassing approach to information provided regarding management of the disease. In order to nurture increased involvement of the employee with DM in self-care and overall disease management, support of the psychological and social impacts of DM must also be addressed (Graffigna et al. 2016:5).

## Purpose and objectives

The purpose of this study was to explore and describe the perceptions of employees with DM, seen by professional nurses in selected occupational health clinics, on the self-management of their disease. The objectives were as follows:

- explore the perceptions of employees with DM visiting occupational health clinics on their self-management practices
- describe the existing support systems for employees with DM in the occupational health clinics.

### Design

A qualitative, exploratory, descriptive contextual design was conducted. The exploratory design allowed for a better understanding of the perception of the self-management practices of the employee with diabetes. The descriptive design focused on the verbatim responses provided by the participants during data collection. Through this approach the interpretation of the data focused less on analysing the information collected and more on a description of the actual perceptions provided by the participant (Nieuwenhuis 2016:54).

### Population

The setting was four selected occupational health clinics within private industries situated in the three subdistricts identified as the Northern, Tygerberg and Western areas. The industries identified were retail, manufacturing and packaging. The accessible population comprised employees (*N* = 55) who were identified as having diabetes (male and female) on the employee register of the four occupational health clinics in Cape Town. The population group interviewed occupied positions from management level to general worker status, the majority of whom belonged to medical aid schemes administered by the workplace. The population was treated by professional nurses in the occupational health clinics in their workplace.

### Sampling

A purposive sampling strategy was used to draw from amongst the accessible employees with DM listed in the registers of the occupational health clinics who met the inclusion criteria for the study. The inclusion criteria for the study was adults with DM, between the ages of 30 and 65 years, permanently employed at the sites selected and registered on the clinics’ databases. The study participants, referred to as employees, were registered by the OHNP at each clinic on disclosure or diagnosis of DM and received support in some manner from the OHNP. The employees had been living with DM for time periods ranging from 3 weeks since diagnosis to 18 years from the time of diagnosis. The criteria assigned to selection were in an effort to gather data that produce ‘rich information’ (Downing 2018:338).

A total of 17 employees were interviewed at the sites (Table 1), after which saturation was reached. Saturation of data was the measure for the number of participants included in the study and was determined once no new information was produced despite new study participants being included (Grove 2017:352).

**TABLE 1:** Number of sites and interviews.

Site	Employees
A	5
B	5
C	2
D	5

### Preparation of the field

An e-mail was sent to the managers of each of the four sites requesting a visit to interview employees, as per the selection criteria, in a private room. The OHNPs, who maintain regular contact with the employees with DM, were asked to contact those eligible employees and request their participation in the study.

#### Pilot interview

A pilot interview was conducted at one of the sites. A semi-structured questionnaire was used to guide the interview with questions posed relating to four areas of focus. These areas focused on the demographics of the employee, how having DM made the employee feel, the employee’s practices in relation to the self-management of DM and the support that the employee received from family and the professional nurse. The semi-structured questions appeared to be appropriate for obtaining the employee’s perceptions of his or her self-management practices as well as the support that the employee received in the occupational health clinic. The data of the pilot interview were therefore included in the data analysis of the main study.

### Data gathering

Individual interviews were conducted in a private and quiet room, as provided by the OHNP at each of the four sites visited. The interviews lasted no more than 40 min each. It was anticipated that the use of the employee’s own workplace to conduct the interviews would allow the employee to feel comfortable sharing his or her ‘story’ (Creswell & Creswell 2018:98). The employees interviewed were not known to the researcher. Prior to commencing the interviews, the study purpose and the process of the interview were explained. The researcher obtained written informed consent from the employees to participate. Permission was obtained from each employee to digitally record the interviews, and a digital recorder was used together with the interview schedule. Field notes were also taken.

### Data analysis

Open coding was used to analyse the data obtained during the interviews. Open coding is described as whittling the data acquired into practicable portions, thus enabling analysis (Gray 2017a:271). To facilitate the process of open coding of the data, the semi-structured interviews were recorded as verbatim transcriptions. The interviews and field notes were analysed together. A detailed analysis of the transcription of the recordings was carried out. The researcher and independent coder acquired a sense of the complexities of the interviews by reading through one transcript to understand the underlying meanings. Short notes were recorded in an attempt to define the underlying meanings of the words whilst reading through the interview. The process was repeated with the remaining transcriptions of interviews. A list of all topics was compiled, clustering together similar topics, and codes were added next to appropriate segments of the text. The most descriptive words of topics were converted to categories. This grouping of the data allowed for the emergence of themes, categories and subcategories. Consensus was reached between the researcher and the independent coder on the final analysis of the data.

### Ethical considerations

This research protocol was approved by the Ethics Committee of the Cape Peninsula University of Technology (CPUT/HWS-REC2015/H09). The study participant has the right to privacy, and all interviewed were reassured that confidentiality of the information shared during the interviews would be maintained (Brink, Van der Walt & Van Rensburg 2018:29). Only the researcher, supervisor and independent coder had access to the findings, which were stored electronically on a password-protected device and the notes and digital recordings in a locked safe. Anonymity was ensured in that code numbers were allocated to each employee, ensuring that there was no link in terms of the employee’s identity and individual responses. No names appeared on the transcribed interviews. Employees had the right to withdraw from the study at any time without fear of recrimination. The researcher followed the interview schedule as compiled and treated each employee fairly during engagement (Gray 2017b:172).

### Measures of trustworthiness

Credibility within the study was demonstrated by the use of the verbatim quotes of the participants. Literature reviewed resonated with the current study results indicative of dependability. Confirmability was established through several of the participants expressing similar perceptions or experiences in relation to their self-management practices. Transferability was identified through the applicability of the study findings referenced in previous studies to the outcomes of the current study.

## Results

### Participants

The sample of participants (*n* = 17) consisted of men (*n* = 13) and women (*n* = 4) between the ages of 30 and 65 years, employed at industries where an on-site occupational health clinic was managed. Participants in this study were referred to as employees. The greater number of male employees in the fields of work included in the study was congruent with statistics indicating that the workforce of South Africa is still more amenable to men than to women (Statistics South Africa 2018:n.p.).

Four main themes emerged from the analysis of the data. These were (1) varied unpleasant conditions and symptoms during the course of the disease, requiring self-management; (2) modification of lifestyle in adapting behaviour to manage the condition; (3) appropriate medication for management of DM and (4) overall support to the employees managing diabetes. This article focused on the theme related to the modification of the lifestyle of employees with DM, including the four categories thereof.

### Theme: Modification of lifestyle in adapting behaviour to manage the condition

**FIGURE 1:**
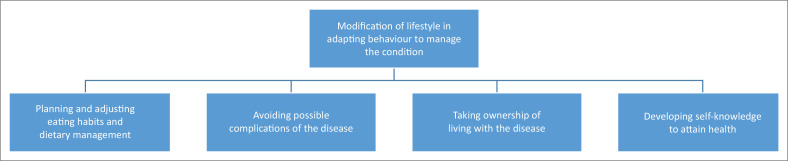
Modification of lifestyle in adapting behaviour to manage the condition.

To motivate a change in behaviour, an individualised and all-encompassing approach to the health education of employees with DM that is promotive of teaching skills for day-to-day living is advocated (Powers et al. 2015:71). These adaptations include physical as well as psychological adjustments. Four categories emerged from the theme of modification of lifestyle, as depicted in Figure 1. These were (1) planning and adjusting eating habits and dietary management, (2) avoiding possible complications of the disease, (3) taking ownership of living with the disease and (4) developing self-knowledge to attain health.

Overall, the employees’ engagements with the recommended changes to their lifestyles were diverse. All employees were aware of the need to adopt a new behaviour, but their experiences of these adaptations varied. An employee expressed a feeling of surprise at her own response to dietary modifications suggested:

‘I don’t really miss it that much. Ha ah “nogal.” It’s actually strange and it’s just a few months from last year that the doctor diagnosed me as diabetic. And I’ve easily, come to think about it, it’s for me even like luxuries stuff. Chocolate stuff. I’m not first in the line anymore.’ (P5, female, site A)

When probed about exercising regularly, an employee’s response was that although aware of the recommendation, there was not sufficient time to do this:

‘I didn’t try exercise yet, because uhm, my time is such a rush and there is no time for me like to do it. My wife is working 7–7 then I must go drop her, pick her up, then the time is not there. And when do I have time to spend with them?’ (P7, male, site B)

Another employee responded positively to an enquiry whether suggested changes regarding self-management made a difference to his health but admitted that maintaining these changes was necessary:

‘I just need to change a couple of things. I’m not exactly where I want to be. I was there and I went backwards and now I’m trying to get back.’ (P15, male, site D)

The findings indicated diverse perceptions of employees on changes that were needed for their lifestyles to take ownership of self-management of the disease. The first step in managing your own meals should involve active planning for healthier eating options and adjustment of incorrect eating habits.

#### Category 1: Planning and adjusting eating habits and dietary management

Implementation of recommended changes to diet requires adjustment and modification in planning meals. Employees expressed some disappointment at not being able to indulge in past food pleasures. An illustration thereof were the following responses when questioned about dietary adaptations:

‘Ja, it is difficult, now I must leave my salt and sauces, everything, like salt and everything. And it is a bit hard… But I must get used to it for my health.’ (P7, male, site B)

‘I just adapted to it. I just tried to eat the right food, change my diet. I am a bit naughty. But like I said, it’s not easy.’ (P15, male, site D)

It was reported that although one was aware of the need to adhere to a healthy diet and self-care, there were occasions when one did not:

‘Sometimes I buy a chocolate (laughing), because you feel like eating something different and breaking away from the normal routine. But if I eat that, I try and eat it once a week and don’t eat it every day. I try to stay disciplined.’ (P2, male, site A)

An employee seemed to accept that a dietary adaptation was necessary, but this appeared to be easier for him to plan for when considering what to eat, as he was not responsible for his own meal preparation:

‘And eating healthy, my mother makes healthy food because it’s me and my father that is diabetic in the house.’ (P14, male, site D)

Some employees commented on being able to find healthy food options when planning their meals but not always being able to afford them:

‘Ha they are not difficult to find, not difficult to find. It’s only the thing that is difficult is about the money, I can’t afford.’ (P11, male, site C)

However, another employee experienced difficulty (field note: swearing) when planning to search for and purchase healthy foods, both in locating the recommended food options as well as financial constraints related to these options:

‘No not really and when you find it, it’s expensive.’ (P17, male, site D)

Adapting bad eating habits and adjusting meals to a well-balanced diabetic way of eating contribute towards avoiding complications of the disease.

#### Category 2: Avoiding possible complications of the disease

Adu et al. (2019:15) state that self-management practices were implemented most often out of concern or fear of developing complications related to DM.

The employees responded to enquiries about which areas of their bodies concerned them most since having diabetes. Responses first related to vision not improving:

‘My eyes, that was actually one of the first signs, visual. I couldn’t see properly. They gave me trifocals and multifocal it didn’t help.’ (P12, female, site C)

‘Eyes, they call it floaters… So I went to the doctor there, uhm, my doctor, uhm is a eye doctor he explained to me what it’s like the cover of our eye, all that sort of stuff …’ (P17, male, site D)

Some of the male employees expressed concern with their genitals since being diagnosed with diabetes:

‘I sometime have problems with him [*penis*]. You know and sometimes when I wee. And it’s only sometimes it’s alright and it feels almost like a yo-yo.’ (P3, male, site A)

‘Uhm now, it’s my penis. Uhm I don’t know why, what happened, but frankly it won’t go over, if I can say it like that, anymore. So I need to go schedule me an appointment for circumcision.’ (P14, male, site D)

A further area of concern was the fear of developing problems with their feet. A concern was:

‘And my feet. And being on my feet all night. It was so, so sore. I would lie awake in bed all night. Even if I haven’t been to work for two days it would still ache. It felt like walking on marbles.’ (P12, female, site C)

One of the employees expressed concern about a cardiac complication and the subsequent need to take care of herself in this area:

‘And also my heart … I have got arrhythmia so I have to be very careful.’ (P4, female, site A)

Managing one’s daily lifestyle and avoiding possible complications of diabetes are indicative of a person taking ownership of managing his or her chronic condition.

#### Category 3: Taking ownership of living with the disease

The results of a study conducted by Jaworski et al. (2018:169) confirmed that the ability to appropriately accept the presence of the disease influences the adherence to dietary recommendations, albeit in a minor way. An employee acknowledged that accepting responsibility for his self-management practices was necessary to delay complications of the disease in order to be present for his partner:

‘I must look after myself and it’s not for me, it’s for my spouse. So that I’m gonna be left here, type of thing. It’s not to be selfish and just stuff myself with whatever.’ (P9, male, site B)

Another employee reached the conclusion that without personal commitment and adhering to dietary recommendations, a loss of productivity could be the result:

‘If I don’t look after my diet, I can feel very tired, really restless and that affects work.’ (P11, male, site C)

An employee alluded to the adoption of new behaviour and resultant changes in lifestyle that were necessitated with the diagnosis of diabetes:

‘It’s not a good thing, I mean because you have to adapt to a certain lifestyle or whatever that I wasn’t used to before.’ (P14, male, site D)

Making lifestyle changes and managing oneself as a person with diabetes will build knowledge about and insight into the vast number of sources available to assist one to be healthy.

#### Category 4: Developing self-knowledge to be disciplined in attaining health

It is anticipated that as knowledge about the disease and the lifestyle adjustments develops, so will an investment in practices that are healthy. With reference to self-discipline and variation regarding diet, rationale was provided for a repeatedly poor eating cycle:

‘Its lack of self-control. This one little cheat will come another one and another one. I feel also tiredness as well, get lazy at work like in December. You’re like ‘let me just have something quick’ and by the third day your body is so tired of eating badly, you have something quick again because you don’t have the energy to cook.’ (P12, female, site C)

Conversely, an employee spoke of his efforts to adhere to an eating plan to try to maintain healthy habits:

‘When I got diagnosed, I got an eating plan from the dietician that I stick to. Uhm, but I try and stay healthy by eating vegetables and eating low GI bread.’ (P2, male, site A)

An employee summarised a commitment to maintaining health through exercise and medications as follows:

‘Yes, I work a lot and I jog a lot and I use the pills the doctor gave me.’ (P13, male, site D)

In the current study, employees sought to know more about their chronic disease, DM, with the anticipation that having more knowledge regarding management of diet and other aspects of the disease would improve their health:

‘I just want to know more about that. Tell me how much sugar and so. I do research but I still would like to know about that perhaps. Maybe if I could get a list.’ (P1, male, site A)

‘… [*B*]ecause diabetic is a kind subject that a lot of people think they know enough and they pass that misconceptions onto me, they really don’t know enough, but I must know.’ (P5, female, site A)

Knowledge about the disease also seemed to bring a measure of acceptance:

‘I know quite a bit, but there is always little things that you learn along the way.’ (P12, female, site C)

It was noted that insight into the condition was gained through communication with others:

‘Uhm, at first you don’t know what to expect, but then I did lot of study about it, I went to go and see people … And I, so now I know what it is.’ (P17, male, site D)

## Discussion of findings

Support should be provided for the patient with diabetes at diagnosis; after that, support should be provided at least yearly, when any complications or co-existing conditions are present or may develop and when there are changes in care (ADA 2018:538). In practice, this care should be constant. In order to improve self-management strategies of those living with diabetes, defining what influences and contributes to behaviour, both positive and negative, is of value. Studies show that there are multiple reasons for patients with DM to not adhere to self-management practices. In this study, it was indicated that the lack of time, challenge of maintaining changes and a feeling of accomplishment were aspects playing a role in changing the employee’s self-management of the disease. Lack of knowledge, limited resources, literacy issues and poor support systems are some of the reasons cited by Mikhael et al. (2019:16) and Reyes et al. (2017:10) acknowledging that lifestyle changes are challenging.

Planning meals and adjusting eating habits were problematic due to various constraints. Several employees in this study noted that the cost of recommended food should be considered in relation to their means. Either they did not have money, or food was unavailable or too expensive or buying higher quality food was impossible. A meta-analysis by Rao et al. (2013:15) found that healthier food options were more expensive. The findings indicated the need for dietary management through planning meals and adjusting eating habits as a necessity in self-management supporting steps. Patients in a study by Reyes et al. (2017:5) communicated that they missed eating some of the food they should not be eating. Substituting healthy options in place of less healthy options was noted as a way of addressing the temptation (Reyes et al. 2017:5).

A shared constraint for participating families is the lack of availability of recommended healthy food options in the marketplace (Jaworski et al. 2018:167). Similarly, individuals were restricted in what they could eat, for example, at social events suitable food options were not being offered (Reyes et al. 2017:4). A study indicated that variations from recommended eating patterns were more likely to occur when the food options suggested deviated from those regularly consumed by the study participants (Jaworski et al. 2018:167). There is a need to identify the factors that may influence the diet of a person with diabetes in a positive and ongoing manner to promote glycaemic control (Burch et al. 2019:1).

The findings indicated that certain complications existed around having diabetes. Two of the serious effects focused around the eyes and the feet. Vision posed a problem amongst patients with DM. The WHO cites the risk of uncontrolled diabetes developing into complications, specifically blindness, kidney failure, lower limb amputation and several additional long-term consequences affecting the quality of life (WHO 2016:30). With regard to the feet, the importance of exercise in the presence of peripheral neuropathy should be emphasised and moderate-intensity walking recommended to counter the progression of this complication (ADA 2018:544). The complications around sexual activities and cardiac diseases were also implied. These also require inputs from both the medical specialist and the patient, but achieving and maintaining optimal personal health is essentially driven by the patient (Raghupathi & Raghupathi 2018:22).

Employees should take ownership of being disciplined in living with diabetes. Individuals could benefit from additional information about their medication, exercise and management of their stress. The SEMDSA made guidelines available for taking ownership of dietary management of patients with Type 2 diabetes (Birkenshaw, Nel & Walsh 2018:39). The ability to appropriately accept the presence of the disease could influence the adherence to dietary recommendations and self-management practices.

Employees felt tired during the workday. A study found that a greater percentage of individuals with Type 2 diabetes experienced symptoms of struggling to rise in the morning and becoming active than those who did not (Thiyagarajan & John 2017:45).

Employees pointed out that development of self-knowledge was needed to maintain a healthy body. The promotion of a positive mindset towards managing the chronic disease is an important step in achieving optimal health outcomes (DuBois et al. 2016:6). Whilst expressing feelings about having diabetes, the participating employees equated knowledge about and a better understanding of the disease with past health experiences. Although newly diagnosed individuals with diabetes were satisfied with the content that education provided for them, those living with the disease for several years experienced the information available to them lacking in terms of their expectations (Dehkordi & Abdoli 2017:115). Recent research regarding the use of diabetes apps suggests that engaging with these resources has the potential to improve self-care and self-management practices, which in turn could result in better overall health (Ayre et al. 2019:8; Kebede & Pischke 2019:12).

Further steps can be taken by employees to improve the management of diabetes (Schulman-Green et al. 2016:1470). The WHO recommends National Guidelines and Management Protocols in terms of lifestyle modifications relating to the principles of healthy living, medication, appropriate screening and referral systems (WHO 2016:50).

Challenging the existing ‘one-size-fits-all’ mode of delivery of health promotion for patients with diabetes is a further measure to augment lifestyle adaptations. In support of an individualised health education provision, the manner in which patients define the recommendations for self-care and self-management should be explored rather than the interpretation of these concepts by health professionals (Masupe et al. 2018:52).

### Recommendations

Nursing education and practice within an occupational health clinic should be enhanced by the professional nurse in support of the employees with DM. Formal appointments at regular intervals should be scheduled with employees to share individualised ‘tailored’ information in relation to self-care and self-management behaviour and healthy living adaptation. This will enable continuous monitoring of the chronic condition, prevention of complications, ongoing education and motivational assistance towards sustainable changes in healthy living through self-management practices. The OHNP should monitor blood glucose levels and review self-care practices to assist in maintaining a more work-productive employee and less time being taken off during working hours due to the disease.

Recipients of health information regarding self-management were better able to practice the principles taught and thus attained better results (Gagliardino et al. 2018:29). An increase in self-management activities should be promoted for general well-being and glycaemic control.

Health promotion sessions on diabetes to all staff within the institution could be a positive effort to prevent stigma related to the disease. The National Standards for Diabetes Self-Management Education and Support should be adhered to as it advocates information sharing through support groups (Beck et al. 2017:1413). Group members should be encouraged to engage in activities such as exchange of recipes or group excursions that include exercise and education of peers and colleagues about the disease.

OHNPs should engage with the relevant stakeholders in the workplace to promote accommodation of the needs of employees with DM, for example, availability of healthy meal choices. Access of employees to a venue on the company premises – for example, a gym – for development of a regular exercise routine, could be an important support measure. Employees should also be advised that the side effects of either their medication or symptoms of the disease could at times hinder their exercise routine and their work. The effects of stress and a loaded work schedule should be outlined for all within the workplace, including the employee with DM and employees encouraged to engage in regular exercise.

Other specialists to consider including for consultation at the workplace are a nutritionist, pharmacologist, social worker or psychologist to address the areas relevant to dietary requirements, optimal medication management as well as education in management of side effects and dosage, respectively. In order for self-ownership of the disease to be acknowledged and implemented through self-management, the development of self-confidence in the individual with the chronic disease is essential (Ebrahimi Belil et al. 2018:8). The collaborative input from the specialists consulted could augment the knowledge of the employee with DM and in turn the confidence levels needed for appropriately and efficiently managing the chronic disease.

Further research is recommended to determine the knowledge gaps of the professional nurse in an occupational health clinic in order to facilitate an enhanced service provision within the workplace.

### Limitations

The findings were limited to those individuals who were employed at the time of the study.

## Conclusion

The findings of this study reveal varied perceptions of the application of recommended changes in lifestyle through self-management practices by the employee with DM. For many employees the challenge is not necessarily in implementing suggested changes but in sustaining them on a day-to-day basis. Further constraints to positive self-management practices were related to some employees not having access to healthier food options, either through a lack of availability or through these options not being affordable.

The professional nurse in an occupational health clinic may assist in the modification of the lifestyle of the employees with DM through education, motivation of the self-care practices adopted and monitoring the employee’s health and living conditions. Further studies focusing specifically on the types of support that could be provided for the employee with DM by the OHNP are recommended.
